# Anesthetic for Children

**Published:** 1879-07-20

**Authors:** 


### *ANÆSTHETIC FOR CHILDREN.*

With a large experience in the use of chloroform as an anæsthetic, Prof. Demme arrives at the conclusion to prefer it over all others as an anæsthetic for children. He says, its action is quicker, and more reliable, and in no way more dangerous than that of the others.

In thirty-two cases he produced an anæsthesia with ether, and among those cases, there were eight in which dangerous symptoms occurred, which made it necessary to employ energetic means to revive the liitle patients.

Prof. Demme complains, especially of the long duration of the stage of excitement, and of the severe emesis which frequently took place during, or after the administration of ether. He also mentions many instances, in which bronchitis, and a few in which disturbances of the bowels followed the use of ether.

Bichloride of methylene had been employed in twenty-eight cases; the children took it better than either chloroform or ether, none of the unpleasant complications of either narcosis were observed, but profound anæsthesia, such as is frequently required, could not be produced by it. The chloride of æthyliden—highly recommended as a safe anæsthetic by Dr. Liebreich—was used in twenty cases; while under its influence, a child of eighteen months, had a sudden and severe attack of asphyxia, which made it necessary to resort to the use of artificial respiration.—*Hospital Gazette.*

